# Draft Genome Sequence of *Zhouia amylolytica* AD3, Isolated from Tidal Flat Sediment

**DOI:** 10.1128/genomeA.00327-16

**Published:** 2016-05-05

**Authors:** Baolei Jia, Hyun Mi Jin, Hyo Jung Lee, Che Ok Jeon

**Affiliations:** a Department of Life Science, Chung-Ang University, Seoul, Republic of Korea; b Freshwater Bioresources Utilization Division, Nakdonggang National Institute of Biological Resources, Gyeongsangbuk-do, Republic of Korea

## Abstract

*Zhouia amylolytica* AD3 was isolated from tidal flat sediment at Taean, South Korea. We report here the draft genome sequence of *Z. amylolytica* AD3, which is the first report of a genome sequence of the genus *Zhouia*. The genomic information will provide a better understanding of the physiology, adaptation, and evolution of *Zhouia* species.

## GENOME ANNOUNCEMENT

The genus *Zhouia* was first proposed as a member of the family *Flavobacteriaceae* to accommodate strictly aerobic, Gram-negative, nonmotile, and moderate halophilic rod bacteria (1). Currently, the genus contains only one species, *Z. amylolytica*, which was isolated from marine sediment in the South China Sea, China. We isolated another strain belonging to *Z. amylolytica* (AD3) from tidal flat sediment at the Yellow Sea, South Korea. The genome sequence of strain AD3 will provide fundamental insights into the ecological adaptation and physiology of *Zhouia* species.

Sequences of 7.12 Mb with 142,445 reads and 296.56 Mb with 7,449,148 reads were generated from 454 GS FLX Titanium system and Illumina MiSeq at ChunLab, South Korea, respectively, and the sequences were assembled using the CLC Genomics Workbench 5.5 (CLC bio, Aarhus, Denmark) and Celera Assembler 7.0 (2). The assembled draft genome sequence of *Z. amylolytica* strain AD3 was 3,787,699 bp long, with a 36.7% G+C content consisting of 31 contigs ranging from 1,101 to 971,372 bp in size. The draft genome sequence was submitted to the Prokaryotic Genome Automatic Annotation Pipeline (PGAAP) of the NCBI for annotation. Genes encoding tRNAs and rRNA operons were determined using the tRNAscan-SE (3) and RNAmmer (4) software programs, respectively. The draft genome sequence of strain AD3 contains 3,197 predicted protein-coding sequences and 25 pseudogenes. In addition, 42 tRNA genes coding for 20 amino acids and 4 rRNA genes were identified. The coding density of the draft genome is 90.0%, with an average gene length of 1,000 bp.

### Nucleotide sequence accession number.

The whole-genome shotgun project has been deposited at DDBJ/EMBL/GenBank under the accession no. AYXY00000000.
